# Isolation of *Streptococcus mutans* in the gastrointestinal tract of corpses

**DOI:** 10.1080/20002297.2025.2610096

**Published:** 2026-01-02

**Authors:** Ami Kaneki, Hiroko Oka, Masashi Ogawa, Yuya Ito, Mariko Kametani, Momoko Usuda, Tatsuya Akitomo, Chieko Mitsuhata, Jinthana Lapirattanakul, Masakazu Hamada, Narutaka Katsuya, Takahiro Harada, Takafumi Nagao, Miki Kawada-Matsuo, Kazuhiko Nakano, Hitoshi Komatsuzawa, Masataka Nagao, Ryota Nomura

**Affiliations:** a Department of Pediatric Dentistry, Graduate School of Biomedical and Health Sciences, Hiroshima University, Hiroshima, Japan; b Center for Cause of Death Investigation Research and Education, Graduate School of Biomedical and Health Sciences, Hiroshima University, Hiroshima, Japan; c Department of Oral Microbiology, Faculty of Dentistry, Mahidol University, Bangkok, Thailand; d Department of Oral & Maxillofacial Oncology and Surgery, Graduate School of Dentistry, The University of Osaka, Osaka, Japan; e Department of Forensic Medicine, Graduate School of Biomedical and Health Sciences, Hiroshima University, Hiroshima, Japan; f Department of Bacteriology, Graduate School of Biomedical and Health Sciences, Hiroshima University, Hiroshima, Japan; g Department of Pediatric Dentistry, Graduate School of Dentistry, The University of Osaka, Osaka, Japan

**Keywords:** *Streptococcus mutans*, gastrointestinal tract, corpses, RNA sequencing, multilocus sequencing typing

## Abstract

**Objective:**

The oral–gut axis, the pathway by which oral bacteria reach the intestine, has recently attracted attention. However, no recent studies have isolated live *Streptococcus mutans*, a major pathogen of dental caries, in the gastrointestinal tract. In the present study, we isolated *S. mutans* from the gastrointestinal tract of corpses.

**Methods:**

Fifty corpses from forensic autopsies (ages 0–94 years, median age 49) were used. Samples were taken from the oral cavity and gastrointestinal tract (esophagus, stomach, duodenum, small intestine, and large intestine) using sterile swabs. *S. mutans* isolates was cultured from the swabs, and DNA and RNA of the bacteria were extracted for genetic analysis.

**Results:**

*S. mutans* was isolated from each organ with the following frequency: oral cavity, 14 cases (28%); esophagus, 3 cases (6%); stomach, 1 case (2%); duodenum, 0 cases (0%); small intestine, 1 case (2%); and large intestine, 4 cases (8%). When *S. mutans* strains isolated from the oral cavity and gastrointestinal tract of the same corpses were compared, the serotypes and genotypes were completely consistent. Bioinformatic analysis showed that gene expression and predicted functions differed between *S. mutans* strains isolated from the oral cavity and the gastrointestinal tract, even though these *S. mutans* strains were the same genotype.

**Conclusion:**

These results suggest that *S. mutans* strains existing in the gastrointestinal tract may undergo changes in gene expression to adapt to the environment of each organ.

## Introduction

Many oral bacteria are swallowed daily along with saliva and food [1,2]. Recent comprehensive analyses of the microbiome have revealed that oral bacteria can reach and colonise the large intestine [3]. This oral–gut axis has attracted attention in the health field [3,4]. The gastrointestinal tract from the oral cavity to the large intestine is physically connected, and understanding the transition of oral bacteria to the gastrointestinal tract and maintaining the health of each organ may become an essential element in protecting the health of the entire body.

The presence of oral bacteria in the large intestine has been demonstrated by recent techniques in comprehensive genetic analysis that do not rely on culture methods [3,5]. This analysis is based on DNA extraction from the patient’s faeces (or intestinal biopsy tissue) and polymerase chain reaction (PCR) using primers targeting specific bacterial species, 16S rRNA gene analysis (next-generation sequencing; NGS), and metagenomic analysis [5–7]. The development of these methods has demonstrated the existence of the oral–gut axis, which had previously been overlooked. The detection of oral bacterial gene sequences from faecal DNA clearly indicates that bacteria originating from the oral cavity reach the large intestine.

Among the oral bacteria detected in the large intestine, periodontal disease-related bacteria such as *Porphyromonas gingivalis* and *Fusobacterium nucleatum* have been the main focus of attention [8]. Oral commensal bacteria, such as *Streptococcus* species, have been detected in faeces by microbiome analysis [9,10]. A paper published in 1980 reported the isolation of *Streptococcus mutans*, a major pathogen of dental caries, from faeces of living humans [11]. However, recent research into the oral–gut axis has paid little attention to the detection of *S. mutans* in the gastrointestinal tract, and no studies have established that live *S. mutans* passes through the gastrointestinal tract and reaches the large intestine.

Identification of bacteria by comprehensive genetic analysis is particularly effective for periodontopathic bacteria, which are often difficult to culture [12]. However, when bacteria are detected by genetic analysis without culturing, the DNA of dead bacteria is also detected, and it is difficult to know whether live bacteria are present [13]. Furthermore, genetic analysis provides only the genetic information of the bacteria, and does not allow the properties of live bacteria to be analysed. Most previous studies have used faeces or biopsy tissue from specific areas of the digestive tract as samples, and no attempt has been made to detect oral bacteria throughout the gastrointestinal tract from the oral cavity to the large intestine. In the present study, we isolated live *S. mutans* strains from the oral cavity, oesophagus, stomach, duodenum, small intestine, and large intestine of corpses, analysed the gene expression, and predicted the function of each *S. mutans* strain.

## Materials and methods

### Ethical approval

This study was conducted in full adherence to the Declaration of Helsinki. This study used residual specimens from swabs and examinations during forensic autopsies. All subjects were forensic autopsies (judicial autopsies and investigative autopsies), rather than pathological or systematic autopsies, and the only contact with the donors was in the autopsy room after death. Therefore, it was impossible to obtain informed consent from the individuals themselves, nor was it possible to obtain parental or legal guardian consent before the autopsy. Therefore, we adopted an opt-out system, disclosing information about the purpose and conduct of the study and ensuring that parents or legal guardians can object to the study’s implementation or continuation. This study was approved by the Hiroshima University Epidemiological Research Ethics Committee (Approval Number: No. E2022-0275), which confirmed the scientific and ethical nature of the research methods and the protection of the human rights of those undergoing forensic autopsies.

### Subjects and samples collection

Fifty corpses that had undergone forensic autopsy at the Centre for Cause of Death Investigation Research and Education, Graduate School of Biomedical and Health Sciences, Hiroshima University, were used. The bodies ranged in age from 0 to 94 years (median age 49) at the time of death, with 27 males and 23 females. The estimated time since death was 1 to 10 days (median time 2 days and 8 h). The estimated time from death to refrigeration for autopsy ranged from 3 h to 156 h (median time 8 h), with 44 of the 50 corpses stored within 24 h. Oral specimens were collected by swabbing the entire tooth surface with sterile swabs (Eiken Chemical Co. Ltd., Tochigi, Japan). For oesophageal and stomach specimens, the mucosal surface was swabbed as widely as possible with sterile swabs. For the small and large intestines, the intestinal tract was partially punctured, and sterile swabs were inserted to swab the mucosal surface within the lumen.

### Isolation and detection of *S. mutans* strains

Swab samples were plated on Mitis–Salivarius–Bacitracin (MSB) agar plates, consisting of Mitis–Salivarius agar (Difco Laboratories, Detroit, MI, USA) supplemented with bacitracin (0.2 U/ml, Sigma-Aldrich Co., St. Louis, MO, USA) and 15% (wt/vol) sucrose, and incubated at 37 °C for 48 h. Colonies with rough colony morphology typical of *S. mutans* strains were picked up from the MSB agar plates and cultured in Brain Heart Infusion (BHI; Difco Laboratories) broth at 37 °C for 18 h. Genomic DNA was extracted from these colonies, and PCR analysis using 16S rRNA primers was performed to confirm the presence of bacterial DNA (Table 1) [14]. Subsequently, PCR analysis using *S. mutans*-specific detection primers was performed using *TaKaRa Ex Taq* (Takara Bio Inc., Shiga, Japan) [15]. The PCR cycle was performed as follows: 30 cycles of 98 °C for 10 s and 70 °C for 1 min. After the PCR products were confirmed by agarose gel electrophoresis using 1.5% agarose gels in tris-acetate-EDTA buffer, the gels were stained with ethidium bromide and viewed under UV illumination. For bacteria that reacted positively with the *S. mutans* detection primers, whole genome sequence analysis was performed at the Genome Information Research Centre, Research Institute for Microbial Diseases, The University of Osaka, to confirm that the strains were *S. mutans*.

**Table 1.** Polymerase chain reaction primers used in this study.

Purpose	Primer	Sequence (5’−3’)	Size (bp)	References
Universal primer	PA	AGA GTT TGA TCC TGG CTC AG	315	[14]
(positive control)	PD	GTA TTA CCG CGG CTG CTG		
*Streptococcus mutans*	MKF-F	GGC ACC ACA ACA TTG GGA AGC TCA GTT	433	[15]
	MKF-R	GGA ATG GCC GCT AAG TCA ACA GGA T		
**Serotype classification**				
Serotype *c*	SC-F	CGG AGT GCT TTT TAC AAG TGC TGG	727	[16]
	SC-R	AAC CAC GGC CAG CAA ACC CTT TAT		
Serotype *e*	SE-F	CCT GCT TTT CAA GTA CCT TTC GCC	517	[16]
	SE-R	CTG CTT GCC AAG CCC TAC TAG AAA		
Serotype *f*	SF-F	CCC ACA ATT GGC TTC AAG AGG AGA	316	[16]
	SF-R	TGC GAA ACC ATA AGC ATA GCG AGG		
Serotype *k*	CEFK-F	ATT CCC GCC GTT GGA CCA TTC C	294	[17]
	K-R	CCA ATG TGA TTC ATC CCA TCA C		
**MLST analysis**				
Transketolase	*tkt*/F	CAG ATT TAT CGG TTA ATG CCA TTC G	751	[18]
	*tkt*/R	TTA GTT GGA GCA CCG TAG CC		
glutamine synthetase type I	*glnA*/F	ACA AAG CGA TGT TTG ATG GCT	631	[18]
	*glnA*/R	GCG TTC TTA CCA TCA CTG CC		
glutamate synthetase	*gltA*/F	TTG AGA CAG ATG CCT GTG GG	564	[18]
	*gltA*/R	AAG CAT GCA GCA TTC CCT TA		
glucose kinase	*glk*/F	AGG GAT TGA TCT TGG TGG AAC A	585	[18]
	*glk*/R	AAA TGA CGT GCA ACA CGG AC		
shikimate 5-dehydrogenase	*aroE*/F	ATG CCT TAC AAG CAG GCA GT	642	[18]
	*aroE*/R	AGC CTG CCA GAT TTC CTG AC		
glutamate racemase	*murI*/F	GAC CTA TTG GTT TTT TAG ACT CCG	520	[18]
	*murI*/R	TCA ATT TTC CCC ACC AGA GGA		
signal peptidase I	*lepC*/F	AGA ATG GGG CCT TTT CTT GGT C	536	[18]
	*lepC*/R	GCC AAA AGC GGA ATT TAA CTT CAC C		
DNA gyrase A subunit	*gyrA*/F	TCG GGC TCT TCC AGA TGT TC	605	[18]
	*gyrA*/R	AGG CGC GAT GTA TAC CCG AT		

### Serotype classification

Serotyping of *S. mutans* strains was performed by PCR using *TaKaRa Ex Taq* with serotype-specific primers for *c*, *e*, *f*, and *k* (Table 1) [16,17]. PCR amplification conditions were as follows: initial denaturation at 96 °C for 2 min, followed by 25 cycles of 96 °C for 15 s, 61 °C for 30 s, and 72 °C for 1 min, and a final extension at 72 °C for 5 min. PCR products were electrophoresed on 1.5% agarose gels, and the gels were viewed under UV illumination after staining with ethidium bromide.

### MLST analysis

The genotypes of *S. mutans* strains were analysed by the MLST method [18,19]. Genome DNA of *S. mutans* strains were amplified by PCR using *TaKaRa Ex Taq* with primers designed on eight housekeeping genes, namely transketolase (*tkt*), glutamine synthetase type I (*glnA*), glutamate synthetase (*gltA*), glucose kinase (*glk*), shikimate 5-dehydrogenase (*aroE*), glutamate racemase (*murI*), signal peptidase I (*lepC*), and DNA gyrase A subunit (*gyrA*) (Table 1). Nucleotide sequences in MLST analysis were obtained using both forward and reverse primers for each housekeeping gene. For all loci except *tkt*, the PCR cycle was performed as follows: initial denaturation at 94 °C for 5 min, followed by 25 cycles (30 cycles for *tkt*) of 94 °C for 30 s, 55 °C (50 °C for *tkt*) for 30 s, and 72 °C for 30 s, and a final extension at 72 °C for 7 min. PCR products were electrophoresed on 1.5% agarose gels, and the gels were viewed under UV illumination after staining with ethidium bromide. The remaining PCR products were purified by illustra ExoProStar 1-Step (GE Healthcare, Little Chalfont, UK) and sequenced using the respective PCR primers. When the nucleotide sequence was unclear, we prepared second sequence samples.

All nucleotide sequences determined during MLST from these isolates were deposited in the GenBank database (accession numbers: PX216560–PX216573 for *tkt*; PX225445–PX225458 for *glnA*; PX225459–PX225472 for *gltA*; PX225473–PX225486 for *glk*; PX225487–PX225500 for *aroE*; PX225501–PX225514 for *murI*; PX236194–PX236207 for *lepC*; and PX236208–PX236221 for *gyrA*). The nucleotide sequences of the housekeeping gene fragments obtained in this study were compared with those deposited in the oral streptococci PubMLST database (http://pubmlst.org/oralstrep/) [20]. The matching results were given the same allele number and sequence type (ST), and the new ones were submitted to the database for designations.

The allelic profiles of *S. mutans* strains were analysed using START (sequence type analysis and recombination tests) [21]. Linkage distances were calculated by determining the number of allelic differences between sequence types (STs) derived from the allelic profiles [21]. Dendrograms were visualised using the unweighted pair group method with arithmetic averages (UPGMA) and the goeBURST (global optimal enhanced based upon related STs) algorithm implemented in the PHYLOViZ programme [22].

### RNA sequencing analysis

RNA sequencing analysis was performed as previously described [23]. *S. mutans* strains was cultured in BHI broth at 37 °C for 18 h. Bacterial cells were lysed using Qiazol (Qiagen, Germantown, MD, USA), and total RNA of *S. mutans* strains were isolated using miRNeasy Micro Kit (Qiagen) according to the manufacturer’s instructions. Library preparation was performed using GenNext RamDA-seq Single Cell Kit (Toyobo, Tokyo, Japan). Whole transcriptome sequencing was performed in 100 base single-end mode using the Illumina NovaSeq 6000 platform (Illumina Inc., San Diego, CA, USA). Sequenced reads were mapped to the reference genome sequence (*Streptococcus mutans* UA159; GenBank Accession: NC_004350.2) using HISAT2 ver. 2.1.0. Counts per gene were calculated using featureCounts v2.0.0.

### Gene set enrichment analysis

Gene set enrichment analysis was performed using *S. mutans* genes that showed two-fold or greater expression changes in the oesophagus or large intestine compared with the oral cavity in the RNA sequencing of each corpse. Gene set enrichment analysis was performed using the ShinyGO 0.82 online resource (https://bioinformatics.sdstate.edu/go/) [23], accessed on 8 July 2025. To determine the genes used in the gene set enrichment analysis, a false discovery rate *p* value cutoff of 0.05 was used.

### Statistical analysis

Statistical analyses were performed using GraphPad Prism 9 (GraphPad Software Inc., La Jolla, CA, USA). Comparisons between two groups were performed using the Man–Whitney U test. For comparisons between three or more groups, the Kruskal–Wallis test was used for nonparametric analysis, followed by the Dunn test for multiple comparisons. Fisher’s exact test was used to compare the detection rates of *S. mutans* strains based on age and sex. Differences were considered statistically significant at *p* < 0.05.

## Results

### Detection of *S. mutans* strains from the gastrointestinal tract

**Figure 1.**
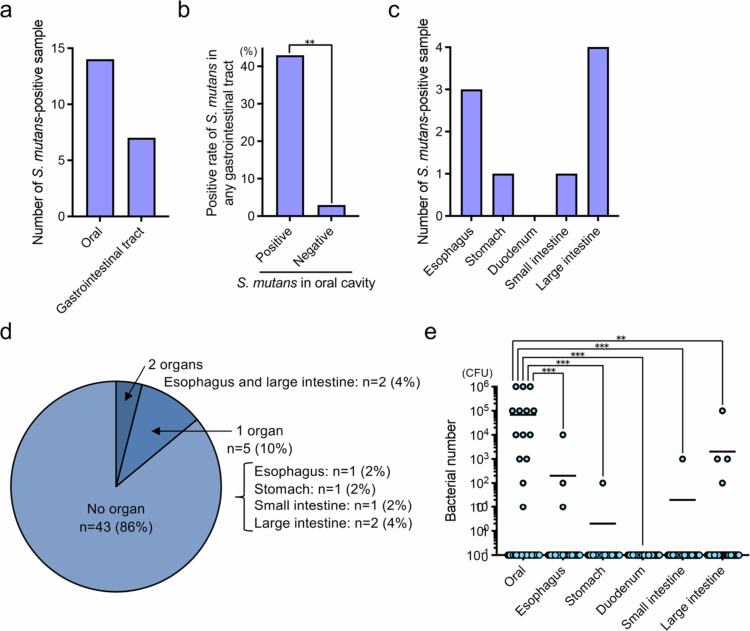
Distribution of *S. mutans* strains in the oral cavity and gastrointestinal tract of corpses. (a) Isolation of *S. mutans* strains in the oral cavity and all areas of the gastrointestinal tract. (b) Correlation between isolation of *S. mutans* strains in the oral cavity and the gastrointestinal tract. (c) Isolation of *S. mutans* strains in different areas of the gastrointestinal tract. (d) Distribution of *S. mutans* strains in the gastrointestinal tract of each corpse. (e) Number of *S. mutans* counts (colony forming unit; CFU) in swabs used to collect samples from the oral cavity and gastrointestinal tract. Each circle indicates one subject. **p < 0.01 and ***p < 0.001 between each group.

Fifty corpses that underwent forensic autopsy to determine the cause of death were included and were numbered DI-001 to DI-050 in the order of arrival. In 14 of the 50 corpses (28%), *S. mutans* strains were isolated from the oral cavity, and in 7 (14%), *S. mutans* strains were isolated from elsewhere in the gastrointestinal tract (Figure 1(a)). There were no significant differences in the detection rates of *S. mutans* strains from the oral cavity and gastrointestinal tract by age or gender (Supplementary Tables 1 and 2). The detection rate of *S. mutans* strains from the gastrointestinal tract was significantly higher in the oral *S. mutans*-positive group than in the oral *S. mutans*-negative group (*p* < 0.01) (Figure 1(b)). Among the gastrointestinal tract specimens, *S. mutans* strains was isolated from three specimens (6%) from the oesophagus, one specimen (2%) from the stomach, none (0%) from the duodenum, one (2%) from the small intestine, and four (8%) from the large intestine (Figure 1(c)). Of the seven gastrointestinal tract specimens in which *S. mutans* strains were isolated, five specimens had *S. mutans* strains isolated from one organ, and two specimens had *S. mutans* strains isolated from two organs (both oesophagus and large intestine) (Figure 1(d)). The number of *S. mutans* counts (colony forming unit; CFU) in swabs taken from each organ was significantly higher in the oral cavity than in the other organs (*p* < 0.01) (Figure 1(e)).

### Serotype classification of *S. mutans* strains

Of the 14 *S. mutans* strains isolated from the oral cavity, 12 were serotype *c*, 1 was serotype *e*, and 1 was serotype *f* (Table 2). All three strains from the oesophagus were serotype *c*, one strain from the stomach was serotype *c*, and one strain from the small intestine was serotype *c*. Of the four strains from the large intestine, three were serotype *c* and one was serotype *e*. The serotypes of *S. mutans* strains isolated from the oral cavity and gastrointestinal tract from the same corpses were all consistent.

**Table 2.** Serotype distribution of *S. mutans* strains.

Sample name	Tissue	Serotype
DI-004	Oral	*c*
DI-006	Oral	*c*
	Small intestine	*c*
DI-009	Oral	*f*
DI-012	Oral	*e*
	Large intestine	*e*
DI-014	Large intestine	*c*
DI-015	Oral	*c*
DI-017	Oral	*c*
	Stomach	*c*
DI-019	Oral	*c*
DI-027	Oral	*c*
DI-030	Oral	*c*
	Oesophagus	*c*
	Large intestine	*c*
DI-033	Oral	*c*
DI-041	Oral	*c*
	Oesophagus	*c*
DI-044	Oral	*c*
DI-049	Oral	*c*
	Oesophagus	*c*
	Large intestine	*c*
DI-050	Oral	*c*

### Multilocus sequencing typing (MLST) analysis of *S. mutans* strains

**Figure 2.**
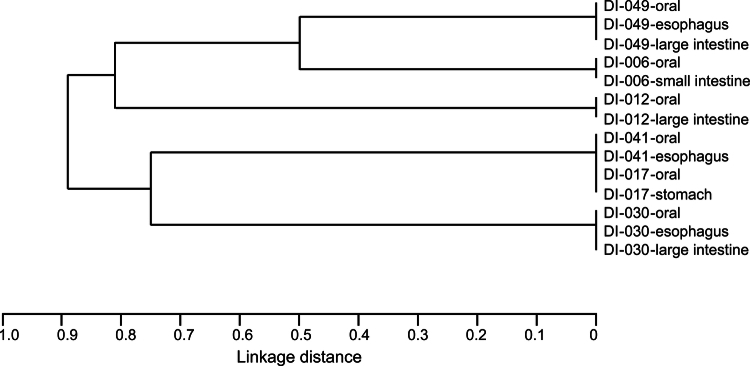
Dendrogram of *S. mutans* strains isolated from the oral cavity and different areas of the gastrointestinal tract. This dendrogram was constructed from allelic profiles of sequence types (STs) detected by multilocus sequence typing (MLST) analysis.

In six samples from which *S. mutans* strains were isolated from both the oral cavity and the gastrointestinal tract, we identified the housekeeping gene sequences and STs of *S. mutans* strains using multilocus sequencing typing (MLST) analysis to compare the *S. mutans* strains isolated from both the oral cavity and the gastrointestinal tract in the same corpse. The housekeeping gene sequences and STs of *S. mutans* strains isolated from the oral cavity and the gastrointestinal tract in the same corpse were completely identical (Table 3) (Figure 2).

**Table 3.** Allelic profiles of *S. mutans* strains in multilocus sequence typing (MLST) analysis.

		Gene								
Sample name	Tissue	*tkt*	*glnA*	*gltA*	*glk*	*aroE*	*murI*	*lepC*	*gyrA*	ST*
DI-006	Oral, small intestine	2	2	53	5	4	5	1	1	394
DI-012	Oral, large intestine	6	1	58	4	5	5	1	4	277
DI-017	Oral, stomach	28	2	49	3	8	28	5	1	252
DI-030	Oral, oesophagus, large intestine	2	3	45	3	11	2	5	4	240
DI-041	Oral, oesophagus	28	2	49	3	8	28	5	1	252
DI-049	Oral, oesophagus, large intestine	14	2	53	1	2	3	1	1	372

*ST stands for sequence type and is defined by the nucleotide sequences of eight housekeeping genes based on MLST analysis.

### RNA sequencing analysis using *S. mutans* strains

**Figure 3.**
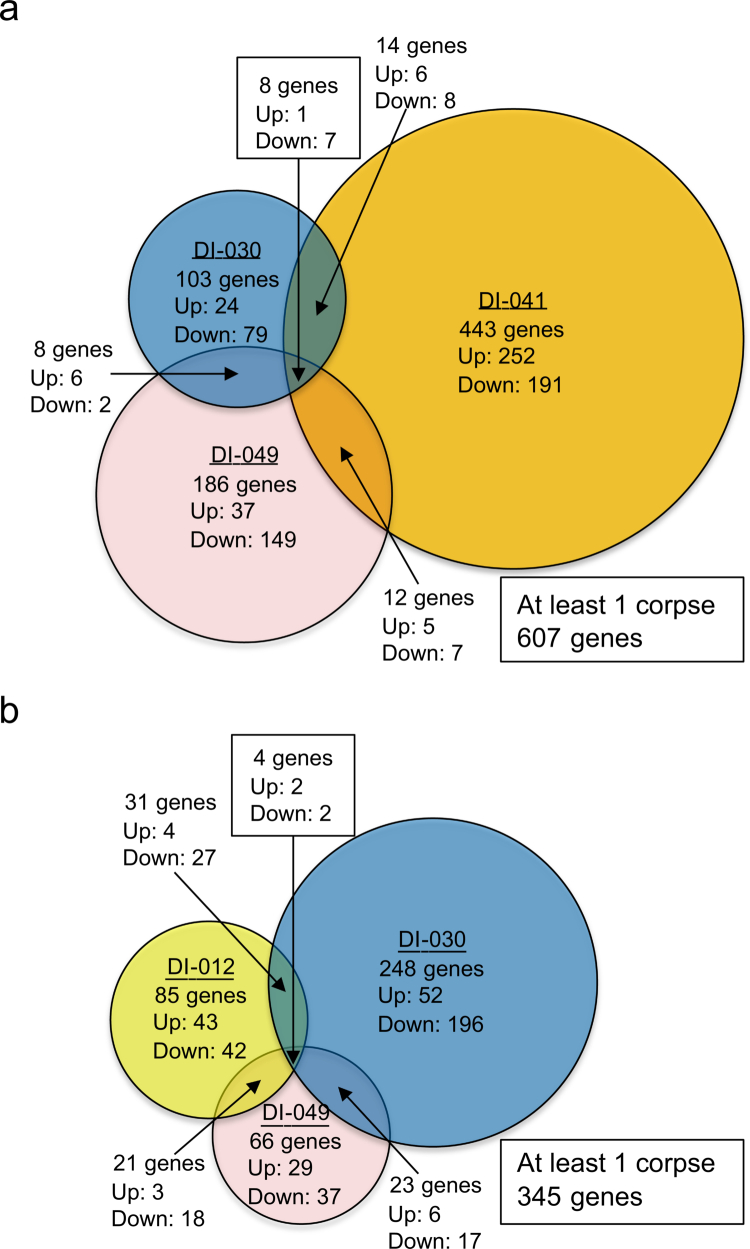
Schematic diagram of RNA sequencing analysis of *S. mutans* strains isolated from the oral cavity, oesophagus, and large intestine of corpses. Genes whose expression levels were up-regulated or down-regulated two-fold or greater in *S. mutans* strains isolated from the oesophagus or large intestine compared with *S. mutans* strains isolated from the oral cavity were extracted. (a) Comparison of oral cavity and oesophagus. (b) Comparison of the oral cavity and large intestine.

Next, we focused on the oesophagus and large intestine, where *S. mutans* strains were isolated from the gastrointestinal tracts of multiple corpses. We used RNA sequencing to analyse the differences in gene expression between *S. mutans* strains that were present in both the oral cavity and the gastrointestinal tract, and which had identical STs in the MLST analysis. We selected genes whose expression levels were up-regulated or down-regulated two-fold or greater in *S. mutans* strains from the gastrointestinal tract compared with those from the oral cavity in the RNA sequencing analysis. In three corpses (DI-030, DI-041, and DI-049) in which *S. mutans* strains were isolated from both the oral cavity and oesophagus, 103 genes (24 up-regulated and 79 down-regulated) in DI-030, 443 genes (252 up-regulated and 191 down-regulated) in DI-041, and 186 genes (37 up-regulated and 149 down-regulated) in DI-049 were up-regulated or down-regulated two-fold or greater in the oesophagus than in the oral cavity (Figure 3(a)). Additionally, 607 genes were found in any of the three corpses, whereas only eight genes (one up-regulated and seven down-regulated) were common to all three corpses (Table 4).

**Table 4.** List of genes whose expression levels were changed by two-fold or greater in *S. mutans* strains isolated from the oesophagus compared to *S. mutans* strains isolated from the oral cavity by RNA sequencing analysis, common to the three corpses. Log₂ fold changes were calculated based on read counts in *S. mutans* strains isolated from the oesophagus/read counts in *S. mutans* strains isolated from the oral cavity.

Gene name	Gene product annotation	D030			D043			D051		
		FPKM		Log₂ fold change	FPKM		Log₂ fold change	FPKM		Log₂ fold change
		Oral	Oesophagus		Oral	Oesophagus		Oral	Oesophagus	
*SMU_1926*	putative transcriptional regulator	12.362	58.538	2.24	0.017	17.724	10.02	0.136	3.677	4.76
*SMU_1928*	putative ABC transporter, permease protein	365.238	179.454	−1.03	226.223	96.405	−1.23	434.114	211.597	−1.04
*SMU_875c*	putative transposase, IS150-like	3.326	1.441	−1.21	2.143	0.000	−1.10	2.427	0.876	−1.47
*SMU_r07*	5S ribosomal RNA	31.092	0.000	−4.96	20.065	0.000	−4.33	20.405	0.000	−4.35
*SMU_r03*	5S ribosomal RNA	46.638	0.000	−5.55	40.130	0.000	−5.32	40.809	0.000	−5.35
*SMU_2076c*	hypothetical protein	63.401	0.000	−5.98	49.097	0.000	−5.61	16.642	0.000	−4.06
*SMU_545*	hypothetical protein	64.268	0.000	−6.00	106.651	0.000	−6.73	132.557	43.546	−1.61
*SMU_t35*	tRNA-Leu	180.600	0.000	−7.50	543.895	0.000	−9.09	158.029	0.000	−7.31

FPKM stands for Fragments Per Kilobase of Transcript Per Million Mapped Reads. FPKM values of zero were adjusted by adding a pseudo-count of 1 prior to calculating fold changes and log₂ fold changes.

In three corpses (DI-012, DI-030, and DI-049) in which *S. mutans* strains was isolated from both the oral cavity and the large intestine, 85 genes (43 up-regulated and 42 down-regulated) in DI-012, 248 genes (52 up-regulated and 196 down-regulated) in DI-030, and 66 genes (29 up-regulated and 37 down-regulated) in DI-049 were up-regulated or down-regulated two-fold or greater in the large intestine compared with the oral cavity (Figure 3(b)). Additionally, 345 genes were found in any of the three corpses, whereas only 4 genes (2 up-regulated and 2 down-regulated) were common to all three corpses (Table 5).

**Table 5.** List of genes whose expression levels were changed by two-fold or greater in *S. mutans* strains isolated from the large intestine compared to *S. mutans* strains isolated from the oral cavity by RNA sequencing analysis, common to the three corpses. Log₂ fold changes were calculated based on read counts in *S. mutans* strains isolated from the large intestines/read counts in *S. mutans* strains isolated from the oral cavity.

Gene name	Gene product annotation	D012			D030			D051		
		FPKM		Log₂ fold change	FPKM		Log₂ fold change	FPKM		Log₂ fold change
		Oral	Large intestine		Oral	Large intestine		Oral	Large intestine	
*SMU_t20*	tRNA-Thr	0.000	651.233	9.35	0.000	59.474	5.92	0.000	180.789	7.51
*SMU_1804c*	hypothetical protein	4.605	14.024	1.61	0.000	7.684	3.12	0.000	11.677	3.66
*SMU_1551c*	putative ABC transporter, ATP-binding protein	20.226	9.269	−1.12	16.494	4.465	−1.88	14.291	3.294	−2.12
*SMU_2076c*	hypothetical protein	100.659	17.588	−2.51	9.347	0.000	−3.23	16.642	0.000	−4.04

FPKM stands for Fragments Per Kilobase of Transcript Per Million Mapped Reads. FPKM values of zero were adjusted by adding a pseudo-count of 1 prior to calculating fold changes and log₂ fold changes.

### Gene set enrichment analysis using RNA sequencing data

**Figure 4.**
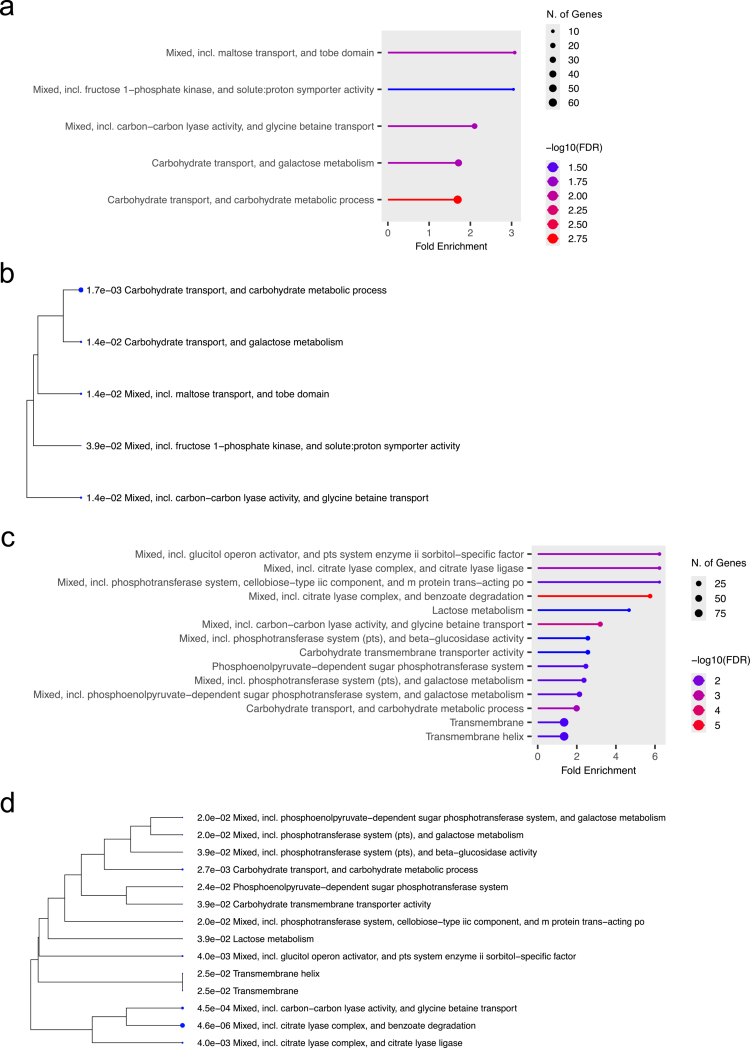
Gene set enrichment analysis using *S. mutans* genes that showed changes in gene expression in the oesophagus or large intestine compared with the oral cavity. Pathways using *S. mutans* genes that showed two-fold or greater changes in expression in the oesophagus compared with the oral cavity (a), and a hierarchical clustering tree summarising the correlations between the pathways (b). Pathways using *S. mutans* genes that showed two-fold or greater changes in expression in the large intestine compared with the oral cavity (c), and a hierarchical clustering tree summarising the correlations between the pathways (d). In the clustering trees, pathways with many common genes are clustered together.

To clarify the putative functions of proteins synthesised from genes whose expression levels, as measured by RNA sequencing analysis, were altered in the oesophagus or large intestine compared with the oral cavity, gene set enrichment analysis was performed using genes whose expression levels were altered in any of the three corpses (607 in the oesophagus and 345 in the large intestine). The results indicated that *S. mutans* strains exhibited altered functions related to carbohydrate (including maltose), proton, and glycine betaine transport in the oesophagus compared with the oral cavity, as well as functions related to carbohydrate (including galactose and fructose) metabolism (Figure 4(a) and (b)). Additionally, *S. mutans* strains exhibited altered functions related to the phosphotransferase system, carbohydrate (including galactose and lactose) metabolism, carbohydrate and glycine betaine transport, transmembrane function, carbon–carbon lyase activity, and citrate degradation in the large intestine compared with the oral cavity (Figure 4(c) and (d)).

## Discussion

In the present study, *S. mutans* strains were isolated from gastrointestinal tracts of corpses, with the highest frequency occurring in the oesophagus and large intestine. The genotypes determined by serotype and MLST analysis were consistent between *S. mutans* strains isolated from the oral cavity and gastrointestinal tracts from the same corpses. RNA sequencing and gene set enrichment analysis demonstrated that gene expression and the predicted functions of *S. mutans* strains from same corpse differed between the oral cavity and gastrointestinal tract. The gene expression of *S. mutans* strains isolated from different corpses differed even among those present in the same organ.

A previous study reported the isolation of *S. mutans* strains from human faeces of living humans [11], suggesting that *S. mutans* is present not only in the oral cavity but also in the gastrointestinal tract. In that study, *S. mutans* strains isolated from faeces were identified by colony morphology under a stereomicroscope [11], and the classification at the time included *Streptococcus cricetus*, *Streptococcus rattus*, and *Streptococcus sobrinus* as *S. mutans*[24]. Nearly 50 years later, little progress has been made in studying the distribution of *S. mutans* strains in the gastrointestinal tract, and its distribution in each part of the gastrointestinal tract has remained unknown. In the present study, we demonstrated the presence of *S. mutans* strains in each area of the gastrointestinal tract by culturing this bacterium and analysing its genes.

*S. mutans* strains were distributed predominantly in the oesophagus and large intestine, but were less abundant in the stomach, duodenum, and small intestine. This result suggests that *S. mutans* strains are present in the oesophagus, which is close to the oral cavity and where bacteria such as *Streptococci* common to the oral cavity can be detected [25,26]. The detection frequency of *S. mutans* strains was low in the stomach and duodenum, where the pH is low and commensal bacteria are scarce [27,28]. *S. mutans* strains were again prevalent in the large intestine, where enteric bacteria are abundant and oral bacterial species are sometimes detected [1]. However, there was no significant difference in the detection rate of *S. mutans* strains in the oesophagus, stomach, duodenum, small intestine, and large intestine. Therefore, more samples need to be analysed to clarify the distribution of *S. mutans* strains in the gastrointestinal tract. In addition, the detection rate of *S. mutans* strains from the gastrointestinal tract was significantly higher in the *S. mutans*-positive group in the oral cavity than in the *S. mutans*-negative group in the oral cavity (*p* < 0.01). The number of *S. mutans* counts (colony forming unit; CFU) in swabs was also significantly higher in samples collected from the oral cavity than in samples collected from other gastrointestinal tracts (*p* < 0.01). These results reaffirm that the oral cavity is the primary colonisation site for *S. mutans* and suggest that the number of *S. mutans* in the oral cavity significantly affects its detection in the gastrointestinal tract.

The PCR analysis using *S. mutans*-specific detection primers, serotype-specific primers, and primers targeting MLST housekeeping genes has been widely used for the identification or classification of *S. mutans* strains isolated from oral specimens such as saliva and dental plaque [15–19].These primers were developed for *S. mutans* strains of oral origin and have not been used to detect *S. mutans* strains isolated from gastrointestinal tract. In the present study, bacteria from the gastrointestinal tract that tested positive for *S. mutans*-specific detection primers, serotype-specific primers, and primers targeting MLST housekeeping genes were identified as *S. mutans* by whole genome sequence analysis. The whole genome sequence contained the identical gene sequences as those obtained with serotype-specific primers and primers targeting MLST housekeeping genes. On the other hand, some strains isolated from gastrointestinal tracts showed amplified bands that appeared to be the correct size with the *S. mutans*-specific detection primers. However, the resulting gene sequences differed from those of *S. mutans*. These strains were negative for serotype-specific primers and primers targeting MLST housekeeping genes, and the whole genome sequence analysis revealed that they contained non-*Streptococcus* species, such as *Enterococcus* and *Klebsiella*. Therefore, to prove that bacteria isolated from the gastrointestinal tract are *S. mutans*, sequence analysis of PCR products with *S. mutans*-specific detection primers or whole genome sequence analysis should be performed. Alternatively, additional PCR analyses using serotype-specific primers or primers targeting MLST housekeeping genes, in addition to *S. mutans*-specific detection primers, are essential.

The detection rate of *S. mutans* strains in the oral cavity in the present study was 28%, lower than the previous detection rates of 36.8% to 72.8% in living Japanese people [29–31]. One possible explanation is that, as oral health in Japan has improved, the detection rate of *S. mutans* in the oral cavity of living Japanese has declined from over 70% around 2000 to approximately 40% in the early 2020s [29,31–33]. Another possible explanation is a lack of food-derived nutrients for *S. mutans* strains in the corpses. *S. mutans* strains derive their nutritional resources from carbohydrates such as sucrose, glucose, and fructose, as well as other fermentable carbohydrates and amino acids, which are primarily found in human food [34,35]. The corpses used in our study were selected from those that had been dead for approximately 1 to 10 days, but it is possible that the lack of nutritional intake during this period limited the nutritional sources for *S. mutans* strains. As a result, the amount of *S. mutans* strains in the oral cavity and gastrointestinal tract was decreased compared with the pre-death levels, which may have led to a lower detection rate of *S. mutans* strains.

MLST analysis is a molecular typing method for identifying bacterial strains and examining their phylogenetic relationships by comparing the sequences of housekeeping genes essential for life [36,37]. MLST analysis revealed that *S. mutans* strains isolated from the oral cavity and gastrointestinal tract of the same corpse were identical in genotype. However, RNA sequencing analysis using these *S. mutans* strains revealed that the expression of 103 to 443 genes in the oesophagus and 66 to 248 genes in the large intestine were altered compared with the oral cavity. *S. mutans* has approximately 2,000 genes [38], and this translates to approximately 5% to 20% gene expression changes in the oesophagus and approximately 3% to 12% gene expression changes in the large intestine. These results indicate that *S. mutans* strains expresses genes differently from those in the oral cavity to survive in the gastrointestinal tract.

In the RNA sequencing analysis, each *S. mutans* strain had only one replicate. Instead, three *S. mutans* strains were isolated from the oesophagus and large intestine of three corpses. We identified specific gene expression patterns of *S. mutans* in the gastrointestinal tract by extracting common genes that showed expression changes in the three *S. mutans* strains isolated from each gastrointestinal tract, compared with those isolated from the oral cavity. Several studies reported RNA or microarray analysis for each *S. mutans* strain using only one replicate, and these studies identified common genes among three *S. mutans* strains exposed to a similar environment [23,39–42]. The previous studies have shown that gene expression can vary significantly depending on the environment, even within the same *S. mutans* strain [23,40–42].

RNA sequencing analysis revealed that among the *S. mutans* genes whose expression changed in the gastrointestinal tract compared with the oral cavity, only eight genes in the oesophagus and four in the large intestine were common across the three specimens. These results indicate that changes in *S. mutans* gene expression in the gastrointestinal tract vary among individuals. The intestinal environment varies greatly among individuals due to genetic factors and lifestyle habits [43], and *S. mutans* strains may not only change gene expression between the oral cavity and the gastrointestinal tract, but may also express different genes depending on an individual’s gastrointestinal environment. Using BLAST and InterPro after RNA sequencing analysis, we identified the genes and functions shared by the three corpses among *S. mutans* genes whose expression levels were altered in the oesophagus and large intestine compared to the oral cavity. The genes whose expression levels were altered in the oesophagus and large intestine were involved in transcriptional regulators (putative transcriptional regulators), transport of substances across the cell membrane (putative ABC transporters, permease proteins, ATP-binding proteins), and translation (5S ribosomal RNA, tRNA-Leu, tRNA-Thr) [44,45].

We performed functional analysis using gene set enrichment analysis for genes whose expression levels were altered in each of the three corpses (607 in the oesophagus and 345 in the large intestine). Gene set enrichment analysis visualises the biological functions and pathways of genes derived from comprehensive gene expression analyses such as RNA sequencing analysis [46]. The gene set enrichment analysis revealed that the oesophagus differed from the oral cavity in the functions related to carbohydrate, proton, and glycine betaine transport as well as functions related to carbohydrate metabolism. The oesophagus has a lower diversity of microbiota than the oral cavity, and is dominated by Gram-positive bacteria such as *Streptococcus* [47,48]. The low bacterial diversity in the oesophagus is because of a lower supply of nutrients to the microorganisms than in the oral cavity [47]. To survive in a nutrient-limited environment, *S. mutans* strains present in the oesophagus may transport and metabolise its main energy source in a manner different from that of *S. mutans* strains present in the oral cavity.

Both the oral cavity and large intestine are organs rich in nutrients and microorganisms. However, the large intestine is dominated by anaerobic bacteria owing to the high nutritional content of digested residues and dietary fibre, and the low oxygen concentration [49,50]. Our results revealed that the function of *S. mutans* strains present in the large intestine differs from that of *S. mutans* strains present in the oral cavity in terms of the phosphotransferase system, carbohydrate metabolism, carbohydrate and glycine betaine transport, transmembrane function, carbon–carbon lyase activity, and citrate degradation, with the phosphotransferase system being particularly prominent. The phosphotransferase system of *S. mutans* functions not only as a sugar transport system involving phosphorylation but also as a global regulatory system controlling the expression of other genes [51,52]. Thus, it is thought that the phosphotransferase system responds sensitively to the colonic environment, which differs from the oral cavity, and it enables efficient sugar transport and environmental adaptation.

In recent years, *S. mutans* has been shown to be associated with the worsening of systemic diseases such as infective endocarditis, cerebral haemorrhage, IgA nephropathy, and ulcerative colitis [53–55]. In the present study, no obvious pathological findings were observed in the gastrointestinal tracts of the corpses from which *S. mutans* strains were isolated. Therefore, it is likely that *S. mutans* strains isolated from the gastrointestinal tract of the corpses resided without directly contributing to systemic pathogenicity. However, *S. mutans* can be pathogenic in patients with underlying diseases or those with impaired systemic function [54,55]. To prevent the worsening of such systemic diseases, maintaining good oral hygiene and reducing the number of *S. mutans* strains in the oral cavity is important to reduce migration of *S. mutans* strains from the oral cavity to the gastrointestinal tract. In future research, we should increase the number of corpses used for analysis and conduct detailed analyses of the distribution of *S. mutans* strains in organs other than the gastrointestinal tract to clarify the impact of *S. mutans* on systemic diseases.

## Conclusions

**Figure 5.**
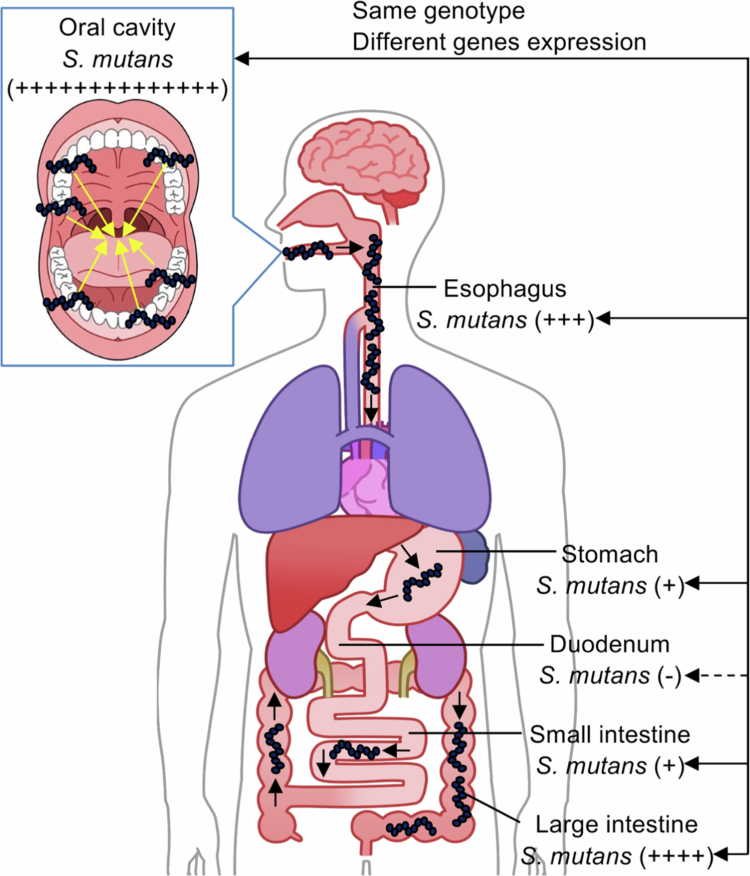
Image showing the distribution of *S. mutans* in the oral cavity and gastrointestinal tract. The number of ‘+’ indicates the number of samples in which *S. mutans* was detected, and ‘-’ means that *S. mutans* was not detected.

*S. mutans* strains were present not only in the oral cavity but also in the gastrointestinal tract of corpses, with a high detection rate in the oesophagus and large intestine (Figure 5). The genotypes of *S. mutans* strains isolated from the oral cavity and gastrointestinal tract of the same corpse were consistent, but the gene expression of these bacteria changed, depending on the type and condition of the organ, to adapt to each environment. Further research is necessary to collect more specimens and clarify the distribution of *S. mutans* strains in each organ.

## Supplementary Material

Supplementary Tables.docx Supplementary Tables.docx

## Data Availability

The data are available from the corresponding author upon reasonable request.
