# Supplementary material for: Isolation of Streptococcus mutans in the gastrointestinal tract of corpses
Source: J Oral Microbiol. 2026 Jan 2;18(1):2610096. doi: 10.1080/20002297.2025.2610096 (PMC12777795; doi:10.1080/20002297.2025.2610096)
Supplement: Supplementary Tables.docx [file ZJOM_A_2610096_SM7070.docx]

**Supplementary Table 1.** Detection of *S. mutans* strains by age.

|  | Age | | |
| --- | --- | --- | --- |
| Tissue | 0-19  (n=8) | 20-59 (n=21) | 60-  (n=21) |
| Oral cavity | 1 (12.5%) | 7 (33.3%) | 6 (28.6%) |
| Gastrointestinal tracts | 1 (12.5%) | 3 (14.3%) | 3 (14.3%) |

There were no significant differences in the detection rate of *S. mutans* strains across organs between age groups, as assessed by Fisher’s exact test.

**Supplementary Table 2.** Detection of *S. mutans* strains by sex.

|  | Sex | |
| --- | --- | --- |
| Tissue | Male  (n=27) | Female (n=23) |
| Oral cavity | 5 (18.5%) | 9 (39.1%) |
| Gastrointestinal tracts | 4 (14.8%) | 3 (13.0%) |

There were no significant differences in the detection rate of *S. mutans* strains across organs between sex groups, as assessed by Fisher’s exact test.
